# Programme Recipient and Facilitator Experiences of Positive Family Connections for Families of Children With Intellectual Disabilities and/or Who Are Autistic

**DOI:** 10.1111/jar.70003

**Published:** 2024-12-19

**Authors:** Daniel Sutherland, Samantha Flynn, Joanna Griffin, Richard P. Hastings

**Affiliations:** ^1^ Centre for Research in Intellectual and Developmental Disabilities (CIDD) University of Warwick Coventry UK

**Keywords:** co‐production, families, intervention evaluation, process evaluation, qualitative

## Abstract

**Background:**

Family members of children with developmental disabilities on average report poorer family functioning and mental health. Positive Family Connections is a co‐produced, positively‐oriented, family‐systems support programme for families of children with developmental disabilities aged 8–13. We investigated experiences of Positive Family Connections, and the processes involved in change.

**Method:**

We conducted semi‐structured interviews with eight family carers who took part in Positive Family Connections and nine facilitators. Data were analysed using framework analysis.

**Results:**

Programme recipients' and facilitators generally reported positive experiences of Positive Family Connections and described beneficial effects on wellbeing and family relationships. We developed a model showing how the lived experience of facilitators and positive approach led to reductions in isolation and perceived changes in mindset that were described as improving family carers' wellbeing and family relationships.

**Conclusions:**

Positive Family Connections appears to be an acceptable programme which programme recipients and facilitators perceive to be beneficial.

## 1 Introduction

Family members of children with developmental disabilities (especially autism, intellectual disabilities or both) are more likely to experience psychological and family difficulties than families of children without developmental disabilities. This includes, on average, elevated rates of mental health problems (Bougeard et al. 2021; Buckley et al. 2020; Rydzewska et al. 2021; Wolff et al. 2022), poorer family functioning (Jackson et al. 2022; Pisula and Porębowicz‐Dörsmann 2017) and lower parental relationship satisfaction (Sim et al. 2016). These findings are also reflected in qualitative research, whereby many parents of children with developmental disabilities describe chronic stress (Nealy et al. 2012), strained family relationships (Downes et al. 2021; Phelps et al. 2009) and frustration and disappointment with services (Griffith and Hastings, 2014; Ryan and Quinlan 2018). However, families often concurrently report positive experiences. Many parents describe how caring for their disabled family member influenced their development as a person or led them to reevaluate priorities in their life (Beighton and Wills 2017). Most family members of people with developmental disabilities do not report clinically concerning symptoms of mental health problems (Hastings 2016), and the increased rates of many difficulties families of people with developmental disabilities experience are significantly attributable to co‐occurring challenges such as poverty (Glidden et al. 2021). It is, therefore, important to challenge simple, negative narratives about the impact of a disabled family member on other members of the family (Hastings 2016). Nonetheless, the elevated rates of psychological and family difficulties indicate the importance of providing families of individuals with developmental disabilities with accessible evidence‐based support.

There is some evidence that family‐systems and peer support‐based interventions may be valuable approaches to supporting families of children with developmental disabilities. Family‐systems interventions conceptualise families as interconnected systems and aim to improve wellbeing by targeting the interactions between family members and their beliefs about family relationships (Dallos and Draper 2015). A systematic review of family‐systems interventions for families of people with developmental disabilities found that, although research was limited and largely of poor quality, family‐systems interventions were typically associated with positive outcomes (Sutherland et al. 2023). Furthermore, in qualitative evaluations of family‐systems interventions, families often described positive effects such as viewing their disabled family member differently or experiencing beneficial changes in family relationships (Sutherland et al. 2023). Similarly, a systematic review of research on peer support interventions for parents of disabled children found that parents valued the opportunity to interact with others who understood their experiences and that peer‐support interventions reduced their sense of isolation, guilt and stress (Shilling et al. 2013). Qualitative evaluations of support programmes which have been delivered or co‐delivered by family carers have highlighted that their shared experiences can aid the development of meaningful relationships, reduce isolation and enhance programmes' credibility (Coulman et al. 2022; Gordon‐Brown et al. 2024; Shilling et al. 2015). A promising approach to support may therefore be to involve family carers in the delivery of family‐systems informed interventions.

Positive Family Connections is a programme aiming to support family relationships and wellbeing in families of children with intellectual disabilities and/or who are autistic and aged 8–13. The focus of the programme on families of children with intellectual disabilities and/or who are autistic is because these conditions are frequently considered together in national policy, as well as both supported through the same health and care services. The programme involves six online sessions which are delivered to groups of 6–8 families, with up to two family carers attending from each family. Positive Family Connections was co‐produced with family carers of children with intellectual disabilities and/or who are autistic to ensure its acceptability and relevance to family carers' priorities, and to incorporate the value of expertise by experience into the programme's development. The programme is also delivered by trained family carer facilitators who are provided with psychological/counselling supervision. In this study of Positive Family Connections, the supervising psychologist was also a parent carer, but this is not a requirement of this role. Each session involves discussion and activities on the overarching themes of a positive approach to family life in families of people with developmental disabilities and a family‐systems‐based approach. Facilitators deliver the programme according to a manual which outlines the key content and activities of each session but are also encouraged to remain responsive to the needs of the group, for example, in how the content is applied and where discussions lead. The programme aims to improve family relationships and wellbeing through peer support and increasing carer's knowledge and awareness of processes leading to positive family relationships and wellbeing. The development of Positive Family Connections, content of the programme, and a logic model describing theoretical assumptions and core change processes are described more fully by Griffin et al. (2023). An outline of the focus of each session, adapted from Griffin et al. (2023), is reported below:

1. Introduction to the programme, family systems, and the positive approach. Discussing each family “Circle of Support” and what makes a positive relationship.
2. ‘Spinning all the plates’ (balancing responsibilities), ‘naming’ difficulties, and managing time. Family carers identify the responsibilities they are juggling and discuss ideas to manage these, as well as discuss time management and prioritisation.
3. Communication, expressing ourselves and active listening. Discuss using the “I” statement and reflect on using active listening skills.
4. Noticing—ourselves and others. Noticing precious moments in family life, family carers' own feelings, and moments of connection with family members.
5. Activities—doing things together, celebrating your family's uniqueness. Group members discuss ways their family spend time together and ways that are different to other families. Families are encouraged to celebrate their own uniqueness.
6. Bringing it all together, tea party, keeping in touch. Re‐capping key messages from the programme and reflecting on what group members are taking away. Discussing how/if group members would like to stay in contact with one another.

A feasibility randomised controlled trial of Positive Family Connections with 60 families has indicated that the programme could potentially be beneficial (Sutherland et al. 2024). Sutherland et al. (2024) found that the programme was delivered with a high level of fidelity and that over 70% of primary parental carers and second carers completed the programme (attending four or more sessions). There was also encouraging evidence that Positive Family Connections was associated with improvements in self‐reported family carer wellbeing and specific family relationships at 9‐month follow‐up. However, a rigorous and comprehensive evaluation of Positive Family Connections should consider qualitative evaluation alongside these quantitative findings.

Qualitative data can inform programme evaluation in several ways. Quantitative outcomes provide data on *whether* a programme was effective but may be uninformative as to *why*. The same programme may produce null findings due to true ineffectiveness, poor implementation, or adverse effects on specific subgroups, and qualitative data can aid in distinguishing between these competing explanations (Skivington et al. 2021; O'Cathain et al. 2015). Second, qualitative data can help researchers to develop a richer understanding of what stakeholders perceive to be beneficial. They can then evaluate this in relation to the logic model outlining the programme's “theory of change”; thereby better understand the mechanisms involved in the programme and inform future development and delivery (Skivington et al. 2021; Noyes et al. 2019; O'Cathain et al. 2015). Third, quantitative outcomes alone may inadequately capture the benefits and harms of programmes (O'Cathain et al. 2015). Participants' experiences of a programme are likely to be complex, value‐laden, and reflective of participants' unique identities and context. Qualitative data can help researchers to characterise participants' perceptions of positive and negative effects of a programme (Skivington et al. 2021). Our research questions for the current study were, therefore:

1. What are programme recipients' and facilitators' experiences of Positive Family Connections?
2. What processes are involved in producing change in Positive Family Connections?

## 2 Methods

### 2.1 Participants

Participants were recruited from a feasibility randomised‐controlled trial of Positive Family Connections (Sutherland et al. 2024). Sixty families were randomly allocated to take part in Positive Family Connections immediately or to a waitlist group. These families included 60 primary parental carers, who the child with developmental disabilities lived with most of the time, as well as 13 s carers, who could be anybody that the primary parental carer considered to be a part of their child's family (but were typically another parent).

The participants were nine facilitators who delivered Positive Family Connections and eight participants who took part in the programme (referred to as programme recipients). All nine facilitators were invited to take part in a focus group or interview, although it was explained that this was optional. Nevertheless, all facilitators agreed to take part. The eight programme recipients were recruited from the pool of 30 primary parental carers and 9 s carers from the 30 families who were allocated to take part in Positive Family Connections as part of the randomised‐controlled trial. We purposively sought to recruit programme recipients with high and low levels of session attendance, and both primary and second carers. However, we were unable to recruit any second carers.

Demographic information for programme recipients is summarised in Table 1. We did not collect demographic data for facilitators given their potential identifiability from previous published outputs from the project. Interviewed programme recipients attended an average of 5.00 sessions of Positive Family Connections compared with an average of 4.03 sessions for all primary parental carers in the Positive Family Connections group of the feasibility study (Sutherland et al. 2024).

**TABLE 1:** Programme recipient information.

Name	Participant information			Family member with developmental disabilities information			*N* of Positive Family Connections sessions attended (from 6)
	Age	Gender	Ethnicity	Age	Gender	Diagnosis	
Sarah	40	Female	White British	8	Male	Autism	6
Rebecca	45	Female	White British	13	Female	Autism	6
Emma	42	Female	White British	12	Female	Autism	5
Katie	48	Female	White British	11	Male	Intellectual disabilities, autism, global developmental delay	6
Maryam	36	Female	Asian/Asian British	8	Female	Intellectual disabilities, autism	5
Aisha	34	Female	Asian/Asian British	13	Male	Autism	2
Chloe	36	Female	White British	8	Female	Intellectual disabilities, autism	5
Laura	42	Female	White British	13	Male	Intellectual disabilities, autism, global developmental delay	5

### 2.2 Procedure

All facilitators and programme recipients were sent a link to an information document about the qualitative study and completed a consent form using Qualtrics. Eight facilitators participated in focus groups (one with four members and two with two members) and one facilitator participated in an individual interview. All programme recipients completed individual interviews with a researcher. Focus groups and the interview with facilitators took place approximately 1–2 months after the final session of the Positive Family Connections programme. Interviews with programme recipients took place after nine‐month follow‐up for the feasibility trial (approximately 5–6 months following the last Positive Family Connections session). Semi‐structured interviews/focus groups covered topics related to programme recipients' and facilitators' experiences of different aspects of the Positive Family Connections programme and perceptions of its effects. The topic guide can be viewed in Data S1A. Interviews and focus groups took place and were recorded using Zoom and were transcribed verbatim by generating automatic transcripts from the Zoom recording, comparing these transcripts against the recording, and correcting any errors. Facilitators and programme recipients were offered a £15 voucher for completing an interview/focus group. This study was granted ethical approval by the University of Warwick Humanities and Social Sciences Research Ethics Committee (HSSREC 57/21‐22).

### 2.3 Data Analysis

Data were analysed using framework analysis, which involves five stages (Ritchie and Spencer 1994). The first stage involved DS familiarising themselves with the data through reading and reflection upon the transcripts. Second, the research team developed a thematic framework for analysis based upon a priori research questions. This was then refined in response to a posteriori themes that were identified during data familiarisation and indexing. The framework included categories related to feasibility questions as well as categories relating to the experiences and impact of Positive Family Connections. The third stage (indexing) involves applying the thematic framework to the data. Indexing was carried out in the software NVivo and was first conducted on three participant transcripts and one facilitator transcript by two independent researchers (D.S. and J.G.). The “coding stripes” NVivo function was used to identify discrepancies between the two researchers, who discussed any differences and reached agreement. D.S. then indexed the remaining transcripts. The fourth analysis stage involves charting—creating matrices with rows for each participant, columns for codes, and each cell populated by brief summaries of the corresponding data. The final stage, mapping and interpretation, involves analytical interpretation of the whole dataset. To ensure that one researcher's meaning‐making did not dominate data analysis, interpretations were shared and discussed with the rest of the research team. Quality was also enhanced by the involvement of the second author (J.G.), who is a family carer as well as a researcher, enabling greater sensitivity to participants' context when interpreting the data (Yardley 2000). A matrix illustrating which participants' responses were related to each of the identified themes can be found in Table 2.

**TABLE 2:** Matrix illustrating which themes were referenced in each participants' responses (*X* = present).

Programme recipients	Value of lived experience of facilitators	Lived experience bolstered peer support	Lived experience added credibility to positive approach	Value of positive approach	Change in mindset	Peer support	Reduced isolation	Improvement in parental wellbeing	Changes in family relationships	Value of programme content	Views on online delivery
Sarah	X		X	X	X	X					
Rebecca	X	X		X	X	X	X	X	X	X	X
Emma	X	X	X	X		X			X		X
Katie	X	X		X	X	X	X	X	X	X	X
Maryam	X	X	X	X		X	X		X	X	X
Aisha	X			X	X	X	X	X	X	X	X
Chloe	X	X		X	X	X		X		X	X
Laura	X	X	X	X					X	X	X
Facilitators											
Olivia	X	X		X		X	X		X	X	X
Hannah	X		X	X		X					X
Julia				X	X	X				X	X
Ben	X	X		X		X	X	X	X	X	X
Robert					X	X				X	
Lara					X	X					X
Nadia	X	X			X	X	X	X		X	X
Sophie	X	X	X	X	X	X				X	X
Millie	X		X	X	X	X				X	X

Framework analysis was selected for several reasons. First, the analysis is guided by both a priori research questions and emergent issues in the data (Parkinson et al. 2016; Ritchie and Spencer 1994). This was appropriate for the current study which involved balancing both specific and relatively concrete questions relating to the feasibility of a future randomised‐controlled trial and partly focused upon more exploratory questions relating to the experiences of Positive Family Connections which are reported in the current study. Framework analysis is also compatible with an experiential focus whilst not requiring the same idiographic, small sample approach as alternative methods such as interpretive phenomenological analysis (Parkinson et al. 2016; Smith, Flowers, and Larkin 2009).

### 2.4 Epistemology

The analysis was grounded in a critical realist epistemology (Madill, Jordan, and Shirley 2000). Critical realism asserts the existence of a “real” (objective/intransitive) natural world. However, access to this is always mediated through (subjective/intransitive) human perception, interpretation, and social construction. Critical realism is characterised by both ontological realism (the belief that reality is independent of minds) and epistemological relativism (the belief that knowledge about reality is relative and that its validity is dependent upon one's circumstances). Critical realist approaches are widely adopted in qualitative research (Clarke and Braun 2013; Madill, Jordan, and Shirley 2000) and were suitable for this research since they recognise the limitations of naïve positivism (which treats human perception as a straightforward and reliable window to knowledge about reality), whilst not discarding with the notion of realist ontology altogether.

### 2.5 Positionality

D.S. and R.H. are White British, male researchers who do not have a family member with a developmental disability. Their understanding of the experiences of families of children with developmental disabilities has therefore largely come from working with families in professional and research contexts. J.G. and S.F. are White British female researchers who have family members with a developmental disability. J.G. is also a psychologist who works with families of children with developmental disabilities.

## 3 Results

**FIGURE 1:**
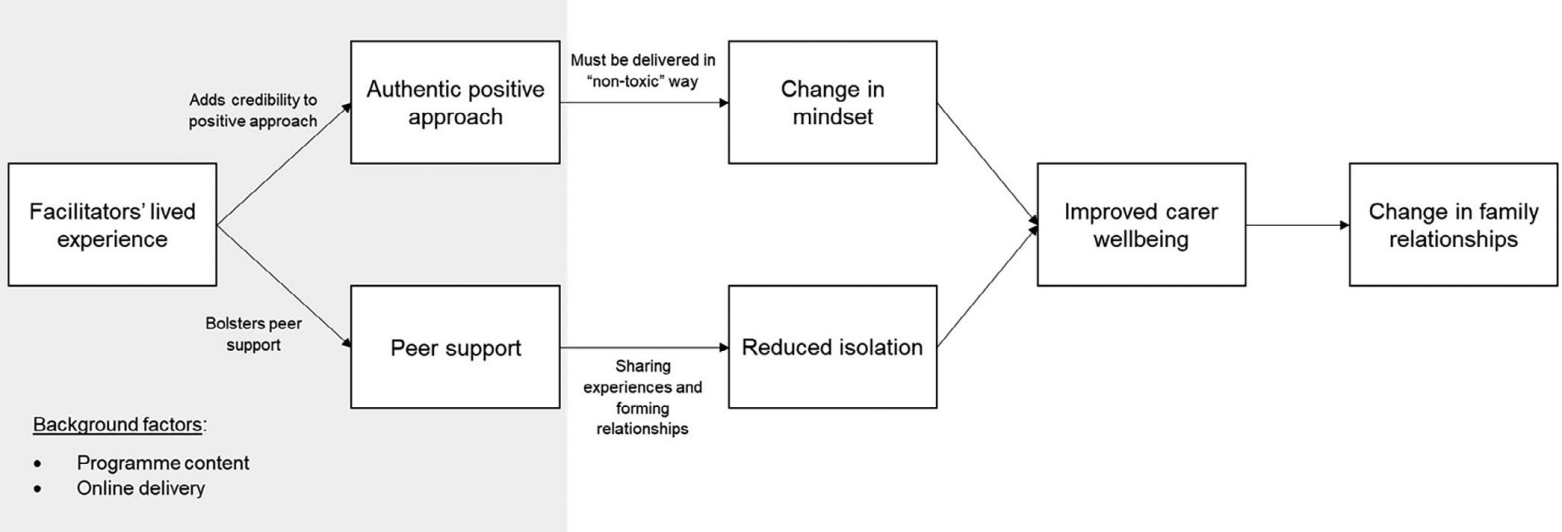
Model of processes leading to change in Positive Family Connections programme.

To summarise the findings, we developed the model displayed in Figure 1. This illustrates the interrelation of key themes discussed by programme recipients and facilitators, and core processes underlying the perceived benefits gained from the programme. The model describes that the lived experience of facilitators was important in two ways: (1) enhancing the peer support function of the programme and (2) by providing credibility to the positive approach. Through shared understanding of experiences, the peer support was perceived to reduce family carers' isolation and build relationships, which in turn had positive effects on programme recipients' wellbeing. Simultaneously, the positive approach, so long as it was delivered in a balanced and reflexive way, was perceived to have beneficial effects on programme recipients' mindsets towards their circumstances which also led to positive effects on carer wellbeing. These positive effects on wellbeing in turn led to downstream changes in family relationships such as effects on parenting, spending time together, and new insights into relationships.

### 3.1 Lived Experience of Facilitators

Programme recipients and facilitators viewed the programme being delivered by family carer facilitators as extremely valuable, particularly by bolstering peer support and adding credibility to the positive approach of the programme.

#### 3.1.1 Bolstered Peer Support

First, facilitators' lived experience bolstered the peer support function of the programme by dissolving boundaries between facilitators and programme recipients and enabling the whole group to bond effectively.

You really felt like they understood and they actually became like, they were part of the group rather than just hosts in a way which felt nice. Rebecca, participant

Facilitators were also able to draw on their personal experiences to inform and contribute to discussion, which in turn helped to create a “safe space” for programme recipients to share their own experiences and for relationships to develop.

There's something about that helping create a safe space…and some of that is perhaps being willing to be vulnerable yourself and to share things about yourself, which allows other people to feel comfortable to tell their story. Olivia, facilitator

They were carers themselves so they got it. They understood the challenges that we face and also they were they were just really approachable. Like you weren't scared to ask a question or speak out. Katie, participant

#### 3.1.2 Adding Credibility to the Positive Approach

In addition to enhancing peer support, facilitators who were also family carers was perceived to bring credibility to the positive approach of the programme. This approach, which was based upon seeking to identify and build upon positive experiences, might have otherwise been perceived as out of touch or dismissive were it to be delivered by professionals without personal experience as carers. However, the perceived relatability of the family carer facilitators equalised this potential power imbalance between facilitators and group members.

It was so much more relatable, so much more relatable and credible, really. And it was refreshing to know that they might be armed with all of this information but some days it just doesn't work you know. Emma, participant

Some of our family carers…they were worried…is it just gonna be like two people who don't really know anything about what it's like to be a carer delivering this content…And, and they felt like, the content was actually really, really good and delivering real life from real life family carers, and still taking up on the challenges as well. Millie, facilitator

### 3.2 Authentic Positive Approach

Most programme recipients perceived the programme's positive approach to be distinctive and valuable. Many reflected that it was easy to become fixated on difficulties and challenges, but they liked having the opportunity to celebrate their families or found a positive attitude to be more pragmatically helpful.

It was nice to actually meet other people and discuss honestly some of those situations but also look positively at how we could improve them. It wasn't just, we're all sitting together and having a moan. Chloe, participant

Programme recipients often explicitly contrasted the positive approach with a “toxic positivity” which was unrealistic or dismissive of difficult experiences. Rather, most programme recipients perceived the programme as being respectful of difficulties that they shared and not dismissive of challenges.

There wasn't any sort of toxic positivity or whatever coming off the facilitators that were very much like, ‘These are difficult sets of circumstances, can we find the positives?’ So yeah, I think it always felt helpful actually, no one was pretending it was kind of all rainbows and you know flowers. Laura, participant

However, there was one participant for whom the positive approach felt inauthentic and unhelpful, highlighting that it may not be welcomed by all carers.

I think always just trying to focus on the positive actually made it feel like that wasn't validating the reality of what our lives were like at that time. Sarah, participant

Facilitators were clearly mindful of the importance of delicately balancing the positive approach with acknowledging and validating programme recipients' difficulties and were sometimes worried about whether they got the balance right.

I think it was hard…to fit the positive approach with sitting with people's pain and difficulty and that was a hard thing to juggle and get right. Hannah, facilitator

### 3.3 Change in Mindset

Some programme recipients described the positive approach leading to changes in perspectives or mindset. The programme helped some group members to recognise everyday, positive experiences that otherwise could be missed, reframe difficulties they were experiencing, and worry less about smaller challenges that were beyond their control. This in turn helped some individuals to feel more present and “take each day as it comes”.

you learn not to sweat the small stuff as much that crops in all the time. So you deal with things as they come more and living more in the moment. Rebecca, participant

It was great to see a kind of shift in mindset from the majority of people and that was really nice to watch. Lara, facilitator

### 3.4 Peer Support

As well as the process described above through which the positive approach led to changes in mindset, there was a second key process relating to group peer support enhanced by family carer facilitation. Programme recipients valued their relationships with group members and the opportunity to share their experiences with others who understood them.

There's not much time I have where I can meet into groups with…families who have similar experiences with children with disabilities and it was nice to like, just talk about those things. It was really, really calming. Aisha, participant

Facilitators similarly described peer support as a critical component of what made the programme valuable, with the content and format of the programme providing the structure for this peer support to develop.

[the] significance of the peer‐to‐peer work to it seems to me that right we've got these staging posts, which is just the structure that we're providing for people, and then they do the work together. And I think that's very powerful. Robert, facilitator

However, one participant was not interested in the peer support aspect of the programme and was instead seeking practical advice and information.

And the sort of social side of things, I can see it's really valuable for some people, but I feel like, well, like we've done that with other groups and we don't need that element. Sarah, participant

### 3.5 Reduced Isolation

The peer support from the programme reduced many carers' sense of isolation. One way that this occurred was practically through the groups providing new opportunities for social relationships, some of which persisted beyond the programme through social media contact and provided long‐term social support.

We've all kind of linked up in a WhatsApp group and are mostly all Facebook friends so we can see how each family is getting on still, and cheer them on and feel like there's somewhere we can go, you know, that, it's a safe space. Rebecca, participant

However, the programme also reduced isolation in a deeper sense through carers feeling less alone in their experiences. For many carers, interacting with others who shared similar experiences and challenges made them feel less isolated or guilty. This helped some carers reframe the difficulties that they or their children experienced as common challenges faced by many families in similar circumstances, rather than feeling like they were inadequate as a parent.

Most of us got really close and learnt a lot about each other's families which really helped you not like feel alone…like there's other people out there that face the same difficulties as we do. So it made me feel like, like I wasn't a failure as a mum. Katie, participant

Nonetheless, there were occasionally challenges in relationships between programme recipients, such as self‐comparisons between group members that disrupted a sense of shared group identity, or the relationships in some groups developing less depth.

I don't think they reached a real depth of connection with this group that I saw with the group that I ran [during piloting]. Ben, facilitator

### 3.6 Improvement in Parental Wellbeing

These two processes of the peer support and the positive approach then converged to positively impact programme recipients' wellbeing. When asked about the impact of Positive Family Connections, programme recipients and facilitators emphasised perceived improvements in their own wellbeing such as feeling calmer or less stressed. This included descriptions of the programme having an immediate positive effect on their wellbeing through enjoyment of the sessions and the “cathartic” effect of discussing their experiences with other carers. However, programme recipients also reported more sustained positive effects on their wellbeing.

I do feel calmer I think in dealing with everything. And try not to deal with everything else, you know, that's unnecessary, I suppose. Yeah, I feel like it's had a positive impact in lots of ways. Rebecca, participant

Programme recipients and facilitators sometimes explicitly linked these perceived improvements in wellbeing with the reduced isolation or change in perspective that the programme provided.

Very helpful because it sort of gave me a different mindset of how I can progress with my difficulties, my mental health…it was nice, just to give that angle that okay, let's not just focus only on the negative we can also focus on the positive and we can take each day as it comes. Aisha, participant

The key messages that that that I hear crop up in, in there around just that ability to share with one another is really important…that, you know, they feel calmer and more relaxed. Ben, facilitator

### 3.7 Changes in Family Relationships

Some carers described the change in their own psychological wellbeing having an effect on their parenting and then leading to downstream positive effects on family relationships within the wider family‐system.

I'm a calmer parent so it's had an impact on my children and, yeah, like I think that positive energy sort of goes to them as well. Rebecca, participant

I think I'm the one main main one who's sort of carrying the baggage. I think, in order for me to function well, then it sort of rolls down and I think if I'm affected in a different way, then it impacts the rest. Maryam, participant

For some programme recipients, there were perceived practical changes in the ways that families related to one another or spent time together.

They'll [participant's children] come and ask mum do you want to play. So before it wasn't like that because they wouldn't think that mum would want to play. Aisha, participant

Other programme recipients described new insights into family relationships that they gained from the programme, such as developing a more systemic perspective, or viewing their family life more positively.

It's one of the first sessions I think where you think about the interdependencies between the children and that was quite profound for me…Because their relationships are not all the same like they've got a really complex set of relationships and drawing my attention to that…was a really important thing for us as a family. Laura, participant

Programme recipients made clear references to perceived changes in their wellbeing leading to reported positive effects on their family relationships. There were also some indications that the reverse influence: changes in family relationships or individuals' perspectives on their family life could also lead to programme recipients feeling more positively about themselves.

I always feel quite, like we'd never do like big things as a family, but it made me realise that things like that are still quality family time and not to not to feel bad about myself because we're not like going to Spain or something. Katie, participant

it's impacted on the child who's got a disability and not just him, but the rest of the children who don't have disability. So I'm a lot more relaxed, more calm. Aisha, participant

However, these reported changes in wellbeing and family relationships could sometimes be difficult to maintain overtime, particularly as new challenges arose.

Very positive while I was part of it, like a really great thing to have been part of. And like many things in life, hard to keep hold of like hard to sort of keep that going. Laura, participant

### 3.8 Background Factors

Whilst there was a key sequence of processes that produced change from Positive Family Connections, there were background factors that interacted with these processes at multiple points and were crucial aspects of the programme.

#### 3.8.1 Programme Content

Many programme recipients did identify particular elements of content that especially resonated with them, or practical tools and exercises that were beneficial for their wellbeing or family relationships. Facilitators also thought that the theory and content made the programme something more than just a parent carer “coffee morning”.

Everybody was able to pinpoint specific things around the content that they felt had been really beneficial. Olivia, facilitator

And some of the meditation type exercises were really nice, just taking a moment. And they were effective, and I do feel calmer I think in dealing with everything. Rebecca, participant

Missing programme content could also disrupt the change processes. For example, facilitators identified that programme recipients missing the second session, which focuses on managing some of the challenges family carers can experience (e.g., managing time and spinning many different ‘plates’), could lead to the positive approach seeming less authentic and helpful in subsequent sessions.

That session is really important. That's the time when…we are owning that there are loads of challenges around bringing up a child with additional needs. …And then you can say right so what are the positive messages in our lives that we can enhance our wellbeing with. And if you miss week two, I think…you get a different message. Julia, facilitator

#### 3.8.2 Online Delivery

The programme being delivered online had positive effects in several ways. For many programme recipients, joining online was more accessible and an in‐person programme would not have been feasible.

I prefer things online. It's really difficult for me to get in or travel somewhere, go to a venue for a few hours. So for me it's easier just to pop in online for two hours. Katie, participant
.

However, some facilitators and programme recipients felt that being online made it more difficult for relationships to develop. There could also be additional challenges for facilitators in online groups; such as re‐directing the focus of discussion or supporting programme recipients who were distressed.

I think it was beneficial that you don't have to do travelling and the extra time that takes. Obviously the negative is because you don't meet people in person it is a little bit harder to sort of build relationships. Chloe, participant

One particular participant who just, I couldn't I couldn't stop her…I think that's the hardest thing of being online…[it] is really tough to bring it back. Julia, facilitator

## 4 Discussion

Programme recipients and facilitators generally had positive experiences of the Positive Family Connections programme and many programme recipients identified ways they believed they had benefited from the programme. These included reduced isolation, positive changes in perspective, and improvements in wellbeing and family relationships. We also developed a model describing the key processes underlying these perceived benefits.

The model we developed is largely consistent with the Positive Family Connections logic model (Griffin et al. 2023). Processes identified in the logic model which were also reflected in interviews included peer support and increased awareness of positive family processes and experiences. Similarly, expected outcomes included increased carer wellbeing and improvements in family functioning. However, the logic model also identifies potential improvements in child behavioural and emotional difficulties as a downstream secondary outcome, something which was occasionally mentioned in interviews but much less frequently. Future quantitative and qualitative work evaluating the impact of Positive Family Connections on child behavioural and emotional difficulties and the mechanisms that may lead to this would be valuable.

The findings are generally consistent with qualitative research on other peer‐support and family‐systems‐based interventions for families of people with developmental disabilities. The description of perceived changes in family relationships from Positive Family Connections paralleled some of those identified in a systematic review of family‐systems interventions for families of people with developmental disabilities (Sutherland et al. 2023). The central role of peer support for family carers is identified in qualitative data from family‐carer‐facilitated group programmes similar to Positive Family Connections, and also in programmes involving one‐to‐one peer mentoring (Coulman et al. 2022; Flynn et al. 2024; Gordon‐Brown et al. 2024; Shilling et al. 2015). These findings highlight the potential common mechanisms of peer‐supported programmes for family carers of children with developmental disabilities, such as the value of mutual understanding and social support. Furthermore, they highlight the value of delivery by individuals with lived experience for enhancing outcomes and programme credibility.

This study has several important practical implications for support for family carers of children with developmental disabilities. First, programme delivery by family carer facilitators was perceived as valuable and core to the programme's change process. Were the programme to have been delivered by non‐family carer professionals, this may have substantially disrupted the peer support function of the programme and the positive approach might have been received much more negatively. The current version of the intervention Logic Model (Griffin et al. 2023) also describes clearly the core role of facilitation by family carers. Given the identified benefits of family carer facilitation, organisations delivering programmes similar to Positive Family Connections might consider involving other family carers as facilitators or co‐facilitators with appropriate training and supervision support. Second, all facilitators and almost all programme recipients perceived the positive approach as valuable. However, both groups were also clearly highly conscious that this would be unhelpful were it to cross into “toxic positivity”. Adopting a positive orientation in support for family carers may be refreshing, but this must be delicately balanced and delivered reflexively in a way that is sensitive to the extremely challenging circumstances of many families of people with developmental disabilities. As we have acknowledged, these families are more likely to experience a range of challenges including poverty (Emerson and Hatton 2007), demanding and stressful care responsibilities (Hatton et al. 2016; McCann, Bull, and Winzenberg 2012), and inadequate support (Ryan and Quinlan 2018; Toms et al. 2015). The potential helpfulness of a positive orientation for some families does not deny these realities, nor suggest that a positive orientation is an alternative to policies and interventions which seek to improve other aspects of families' lives.

There are several strengths and limitations of this research that should be considered. The analysis of qualitative data from both facilitators and programme recipients enables the triangulation of key perspectives to develop an informative model of Positive Family Connections' processes. Through their involvement in developing the programme, the research team have a sophisticated understanding of Positive Family Connections which enabled a rich analysis of programme recipients' and facilitators' data. The involvement of a researcher who is also a family carer (J.G.) in the analysis was also valuable for enhancing sensitivity to family carers' contexts (Yardley 2000). However, despite the research team's attempts to conduct the analysis rigorously and reflexively, it is possible the research team's involvement in developing Positive Family Connections may have introduced biases into the analysis. We also struggled to recruit a sample of programme recipients with a range of experiences within the study as intended. Consequently, interviewed programme recipients were all female primary parental carers and had on average slightly higher levels of programme attendance than all primary parental carers in the trial (5.00 vs. 4.03 sessions). Whilst we did interview one programme recipient who only attended two sessions and referred to other appointments as the cause for non‐attendance, we struggled to gain broader insight into the barriers to attendance and engagement. It is possible that programme recipients with more positive experiences of the Positive Family Connections programme were more likely to respond to interview invitations, and that the current findings reflect a biased perspective. It is also crucial for future research to explore the acceptability and experiences of Positive Family Connections with fathers of children with developmental disabilities, which are not reflected in this research.

There are several ways future research could build upon these findings. Larger‐scale delivery of Positive Family Connections would likely depend upon implementation through other organisations, rather than training and supervision being conducted by the team responsible for developing the programme. Future qualitative evaluation of facilitators' and programme recipients' experiences may assist in identifying new issues that arise during wider implementation. Second, there is a need to obtain data from individuals who had poorer attendance at the programme to better understand why family carers may disengage, more critical perspectives on the programme, and how to support carers in completing the Positive Family Connections programme if they wish to. Third, evaluation by researchers independent of the development of Positive Family Connections would be useful.

## Ethics Statement

Ethical approval was granted by the University of Warwick Humanities and Social Sciences Research Ethics Committee.

## Supporting information


Data S1.

## Data Availability

Data are detailed interviews and focus groups related to potentially vulnerable children and thus are not ethically appropriate for sharing given the risk of participant identifiability.
