# Supplementary material for: Programme Recipient and Facilitator Experiences of Positive Family Connections for Families of Children With Intellectual Disabilities and/or Who Are Autistic
Source: J Appl Res Intellect Disabil. 2024 Dec 19;38(1):e70003. doi: 10.1111/jar.70003 (PMC11658960; doi:10.1111/jar.70003)
Supplement: Supplementary file 1 — Data S1. [file JAR-38-e70003-s001.docx]

**Supplementary materials A**

*Interview and focus group topic guides*

All interviews and focus groups were semi-structured and so the following topic guides were used a rough guide but were supplemented with additional questions to explore in greater detail the comments of facilitators and programme recipients. The interviews also involved some questions related to other aspects of the feasibility of a trial of Positive Family Connections (e.g., acceptability of surveys, randomisation etc.). Since these are not relevant to the current paper, which is focused on experiences of the programme itself, only items from the section of the interviews concerning the programme itself are included below.

**Programme recipient interviews**

- Was there anything that made it difficult to attend Positive Family Connections sessions?
  - Is there anything that might have helped?
  - Were reminder emails each week helpful?
- Can you tell me about your experience of taking part in Positive Family Connections?
  - Specific questions to ask about if the participant doesn’t mention them:
    - How did you find the facilitators?
    - What did you think about having the group led by facilitators who are family carers?
    - How did you find your group members and relationships with them?
    - What did you think about the positive focus and focus on the whole family?
    - How did you find the content of the course?
    - What did you think about the structure of the course and sessions?
    - If applicable: What was your experience of taking part with a second family carer?
- Has Positive Family Connections had any impact on you? What was this?
- Has Positive Family Connections made any impact on the rest of your family? What was this?
  - What about the programme do you think was most important in making a difference to you and your family?
  - Specific questions to ask about if the participant doesn’t mention them:
    - Did you learn any new skills or ideas?
    - Did you gain anything from the other family carers in the group?
    - Did the course affect you individually in any way?
    - Did the relationships with others in the group affect you in any way?
- What did you think was good about Positive Family Connections?
- What would you change?
- Do you think the programme was a good cultural match for you?
- What do you think about taking part in a programme which has been co-produced with family carers?
- What do you think about the number and length of sessions?
- How did you find doing the programme online?
- Have you applied anything you learned from Positive Family Connections? If so, how?
- Was Positive Family Connections different to other support you have received? How?
- Can you think of anything we may have missed to talk about?
- Is there anything you would like to add?

**Facilitator interviews/focus groups**

- How did you find the experience of facilitating the programme overall?
  - What did you think of the content of the sessions?
  - How did you find using the manual?
  - How did you find working with the other facilitator(s)?
  - What did you think of the focus on a positive orientation and a family-systems approach?
- How do you feel group members’ engagement with Positive Family Connections was?
  - Do you think they learned any new knowledge or skills? What were these?
  - How were their relationships with each other and with you?
  - Do you feel the programme had any other impact?
- What do you think was most successful in facilitating the programme?
- What was most challenging?
- Do you feel that you were supported well?
  - What extra support would be helpful?
- What do you think makes a good facilitator for the programme?
  - What should we look for in future facilitators?
- Of all the things we have talked about today what do you think is the most important?
- Can you think of anything we may have missed to talk about?
- Is there anything you would like to add?
